# FAAH Modulators from Natural Sources: A Collection of New Potential Drugs

**DOI:** 10.3390/cells14070551

**Published:** 2025-04-05

**Authors:** Catalin Nicoara, Filomena Fezza, Mauro Maccarrone

**Affiliations:** 1 Department of Biotechnological and Applied Clinical Sciences, University of L’Aquila, Via Vetoio, Coppito, 67100 L’Aquila, Italy; catalin.nicoara@students.uniroma2.eu; 2 Department of Experimental Medicine, Tor Vergata University of Rome, Via Montpellier 1, 00121 Rome, Italy; 3 European Center for Brain Research/Santa Lucia Foundation IRCCS, Via Del Fosso di Fiorano 64, 00143 Rome, Italy

**Keywords:** FAAH, natural compounds, inhibitors, cannabinoids, endocannabinoid system

## Abstract

The endocannabinoid system (ECS) plays a crucial role in maintaining homeostasis by regulating immune response, energy metabolism, cognitive functions, and neuronal activity. It consists of endocannabinoids (eCBs), cannabinoid receptors (CBRs), and enzymes involved in eCB biosynthesis and degradation. Increasing evidence highlights the involvement of the ECS under several pathological conditions, making it a promising therapeutic target. Recent research efforts have focused on modulating endogenous eCB levels, particularly through the inhibition of fatty acid amide hydrolase (FAAH), the main catabolic enzyme of the major eCB anandamide. Natural substances, including plant extracts and purified compounds, can inhibit FAAH and represent a promising area of pharmacological research. Natural FAAH inhibitors are particularly attractive due to their potentially lower toxicity compared to synthetic compounds, making them safer candidates for therapeutic applications. Phytocannabinoids, flavonoids, and flavolignans have been shown to efficiently inhibit FAAH. The structural diversity and bioactivity of these natural substances provide a valuable alternative to synthetic inhibitors, and may open new avenues for developing innovative pharmacological tools.

## 1. Introduction

**Figure 1:**
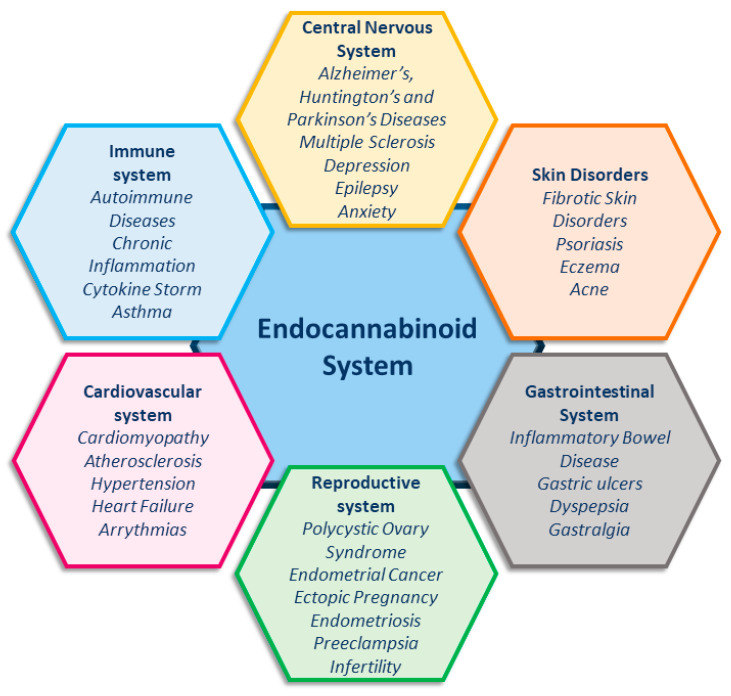
Endocannabinoid system and related pathologies.

The endocannabinoid system (ECS) is essential for maintaining homeostasis, as it regulates the immune system, energy metabolism, and cognitive functions, and also modulates neuronal activity [1,2]. Unsurprisingly, increasing evidence shows the involvement of the ECS in an ever-growing number of pathological conditions (Figure 1) [1,2,3,4,5,6,7,8,9], making this system an important target for developing new therapies in medicine.

**Figure 2:**
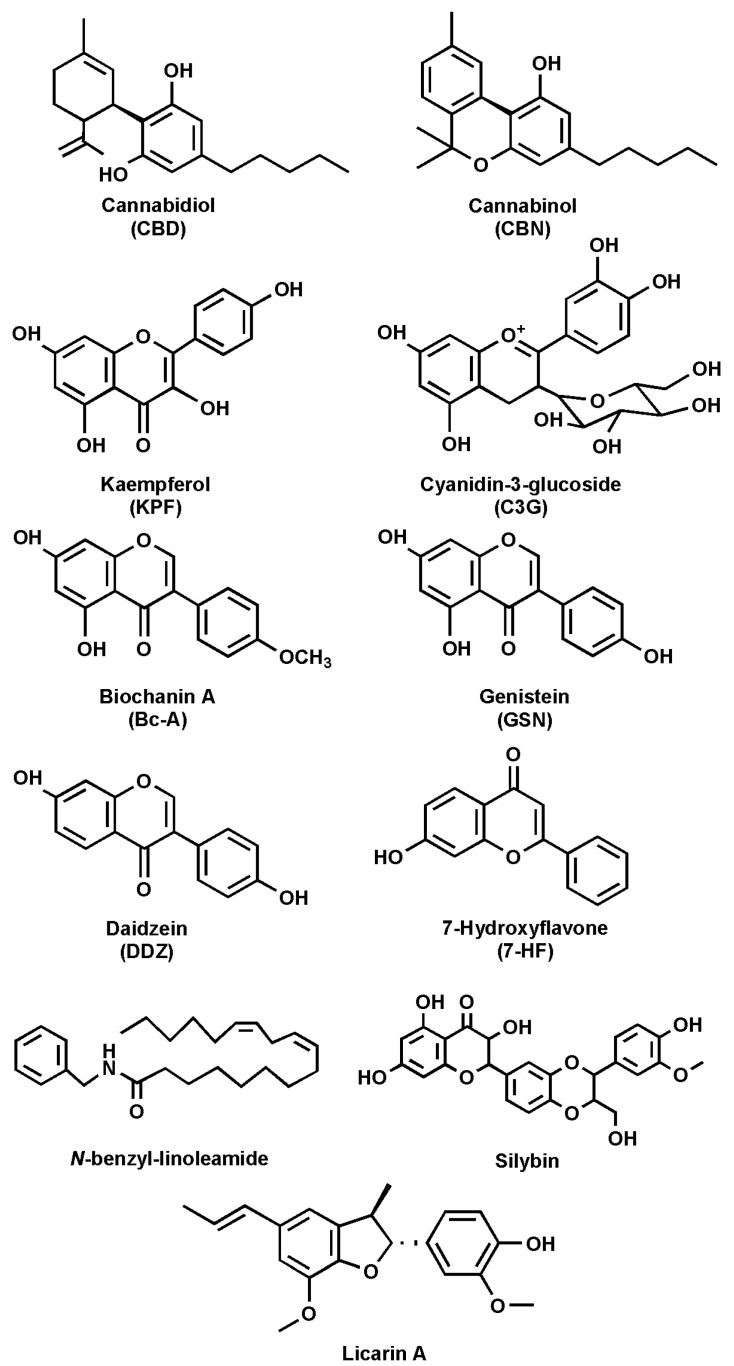
Chemical structures of the main natural FAAH inhibitors.

The ECS comprises endocannabinoids (eCBs), cannabinoid receptors 1 and 2 (CB_1_R and CB_2_R), and the enzymes responsible for eCB synthesis and degradation [10,11]. The main eCBs, *N*-arachidonoylethanolamine (anandamide, AEA) and 2-arachidonoylglycerol (2-AG), are derived from C20:4 ω-6 arachidonic acid (AA) and belong to the *N*-acylethanolamines (NAEs) and 2-monoacylglycerols (MAG) families, respectively [12,13]. Besides AEA and 2-AG, other derivatives of ω-6 and ω-3 polyunsaturated fatty acids (PUFAs) have also been identified, including *N*-linoleoylethanolamine, *N*-eicosapentaenoyl ethanolamine, and *N*-docosahexaenoylethanolamine [14]. The main receptors of eCBs (CB_1_R and CB_2_R) belong to the family of G protein-coupled receptors (GPCRs) [3,15]. Indeed, it was the interest in the pharmacological and psychotropic effects of trans-Δ^9^-tetrahydrocannabinol (THC), one of the two main plant-derived phytocannabinoids (pCBs), that led to the discovery of CB_1_R and, a few years later, of CB_2_R [1,16]. It seems noteworthy that many pCBs, including cannabidiol (CBD) (Figure 2), have low binding affinity for both CBRs [17,18].

In fact, CBD is a negative allosteric modulator with low affinity for both CB_1_R and CB_2_R, yet it also acts on non-CBR molecular targets, such as transient receptor potential vanilloid type 1, 2, 3, and 4; peroxisome proliferator-activated receptor γ; serotonin receptor type 1 and 2, G-protein receptor 55; and adenosine A1 and A2 receptor [2,9,19,20,21,22,23,24]. The third component of the ECS consists of enzymes responsible for the biosynthesis and degradation of eCBs. The biosynthesis of AEA and 2-AG occurs in two main steps. For AEA, the first step involves N-acyltransferase (NAT) enzymes, which transfer an acyl group to phosphatidylethanolamine, forming *N*-acylphosphatidylethanolamine (NAPE). The second step is catalyzed by N-acyl phosphatidylethanolamine-specific phospholipase D (NAPE-PLD), which hydrolyzes NAPE to produce AEA and phosphatidic acid [8,12,25]. Similar to AEA, the biosynthetic pathway of 2-AG can be divided into two distinct steps. First, phospholipase C (PLC) hydrolyzes phosphoinositides like 2-arachidonoyl-phosphatidylinositol, generating 2-arachidonoyl-diacylglycerol (2-Ar-DAG) [26]. Next, diacylglycerol lipase (DAGL) converts 2-Ar-DAG into 2-AG [8,27,28]. Notably, the biosynthesis of 2-AG is tissue-specific due to the selective recognition of different phosphoinositides by PLC [12]. Additionally, there are alternative biosynthetic pathways for both AEA and 2-AG [12]. The primary catabolic enzyme responsible for the degradation of AEA is fatty acid amide hydrolase (FAAH), which produces AA and ethanolamine [28], but also appears to play a catabolic role for 2-AG [12,26]. Several hydrolases are currently known: FAAH or FAAH-1, predominantly expressed in the brain; FAAH-2, found in human adipocytes; and the N-acylethanolamine acid amide (NAAA) hydrolase, which is localized in lysosomes [12]. Additionally, AEA is also catabolized by oxidative enzymes, including 12-lipoxygenase (12-LOX), cyclooxygenase-2 (COX-2), and cytochrome P450 [12].

**Figure 3:**
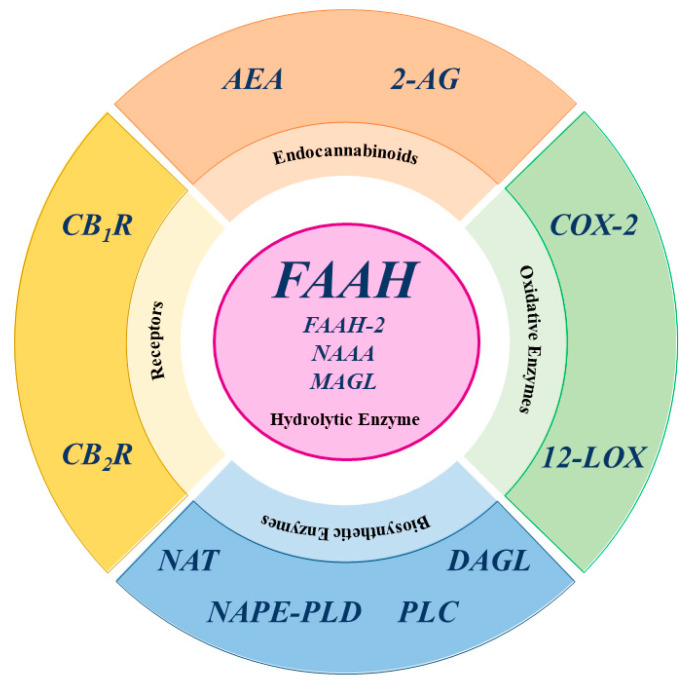
Schematic representation of the main ECS elements. AEA: anandamide; 2-AG: 2-arachidonoylglycerol; COX-2: cyclooxygenase-2; CB_1_R cannabinoid receptor type 1; CB_2_R cannabinoid receptor type 2; DAGL: diacylglycerol lipase; FAAH: fatty acid amide hydrolase; FAAH-2: fatty acid amide hydrolase-2; 12-LOX: 12-lipoxygenase; MAGL: monoacylglycerol lipase; NAAA: N-acylethanolamine acid amide hydrolase; NAPE-PLD: N-acyl phosphatidylethanolamine-specific phospholipase D; NAT: N-acyltransferase enzymes; PLC: phospholipase C.

In contrast, 2-AG is primarily catabolized by monoacylglycerol lipase (MAGL), but also by other enzymes, such as COX-2, 12-LOX, α/β-hydrolase domain-containing 6 (ABHD6), and α/β-hydrolase domain-containing 12 (ABHD12), with FAAH playing a minor role [26,28]. It is noteworthy that MAGL, ABHD6, and ABHD12 exhibit specific distribution within the central nervous system (CNS), suggesting distinct physiological roles in the regulation of 2-AG signaling [12]. A schematic representation of the main ECS elements is shown in Figure 3.

The manipulation of the ECS began through the use of compounds capable of activating the cannabinoid receptor, leading to changes in brain signaling, including undesirable psychotropic side effects [17]. These complicating factors have hijacked research towards agents capable of manipulating endogenous endocannabinoid levels to avoid unwanted adverse effects. Among the various enzymes involved in endocannabinoid degradation, one of the most studied and that has attracted research, also by the pharmaceutical industry, is FAAH.

### FAAH Structure

FAAH is a dimeric enzyme, composed of two identical monomers anchored to the endoplasmic reticulum (ER) membranes, and able to regulate the hydrolysis and biological activity of AEA, as well as of other NAEs and, to some extent, of 2-AG [29]. Each monomer consists of two transversal α-helical structures (α-18 and α-19) and an *N*-terminal transmembrane domain of 20 amino acids (residues 9–29), allowing the substrate to reach the active site though a membrane channel [29,30]. The parallel orientation of the monomers allows both subunits to simultaneously bind and hydrolyze substrates [31]. FAAH is a serine hydrolase situated on the membrane of ER [32]. The binding site consists of three functional channels: the membrane access channel (MAC), acyl binding pocket (ABP), and cytosolic port (CP). Within the active site, the substrate is surrounded by the catalytic triad consisting of Ser241, Ser217, and Lys142, which are essential for the enzymatic reaction. Furthermore, the residues Gly240, Gly239, and Ile238 play crucial roles in substrate stabilization [29,33,34].

FAAH belongs to a large class of hydrolytic enzymes known as the amidase signature (AS) family. However, this family has diverged significantly in terms of substrate specificity and function [35]. Unlike most other AS enzymes, which are primarily soluble proteins, FAAH is an integral membrane enzyme that preferentially binds and hydrolyzes hydrophobic substrates, including amides and esters [29].

Remarkably, FAAH is an allosteric enzyme and represents an interesting case of communication between two enzyme subunits, controlled by a single amino acid at the dimer interface [31]. Additionally, as previously reported for CB_1_R [36,37], the important connection between steroids and the ECS has also been demonstrated for rat FAAH (rFAAH). In particular, the molecular interaction of different steroids with rFAAH appears to be able to modulate the enzyme’s membrane-binding properties, impacting the biological activity of eCBs [38]. Notably, cholesterol, a key sterol in membrane composition, has also been shown to influence FAAH activity by stabilizing its interaction with lipid bilayers and facilitating substrate accessibility to the active site [39].

Based on the current knowledge of rFAAH and human FAAH (hFAAH), a high homology has been identified, with a difference of six amino acids between the two FAAH isoforms [33,40]. Additionally, the secondary and tertiary structures of hFAAH and rFAAH are not identical, showing specific differences around the side chain of the aromatic residues [41].

## 2. FAAH Modulators

FAAH modulators are molecules that can regulate FAAH activity in either a positive or a negative manner; among them, inhibitors play a central role. By blocking enzyme activity, inhibitors increase levels of NAEs, and particularly of AEA, *N*-palmitoylethanolamine, and *N*-oleoylethanolamine, in both the CNS and at the periphery.

Initially, the first inhibitors discovered, such as phenylmethylsulfonylfluoride, trifluoromethyl ketones, and fluorophosphonates, exhibited good inhibitory activity but showed poor selectivity for FAAH [42,43]. In fact, they also interacted with various serine hydrolases (e.g., MAGL), as well as CB_1_R, neuropathy target esterase, acetylcholinesterase, and butyrylcholinesterase [43].

Then, more selective inhibitors were identified and subsequently classified based on their action mechanism. Specifically, the three main categories include irreversible covalent inhibitors, reversible covalent inhibitors and reversible non-covalent inhibitors (Table 1) [42].

**Figure 4:**
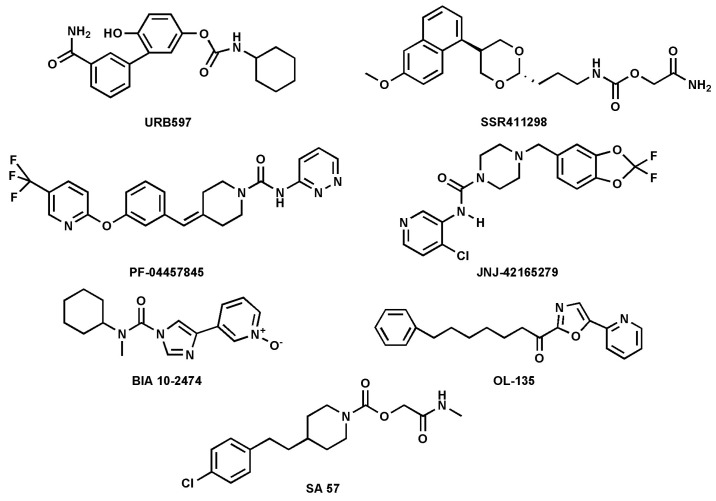
Chemical structures of the synthetic FAAH inhibitors listed in Table 1.

FAAH inhibitors contain electrophilic groups, such as carbamates, urea, and ketoheterocycles, which react with the catalytic site, forming new bonds with the enzyme (Table 1 and Figure 4 [44,53].

Carbamates are potent FAAH inhibitors *in vivo*, due to their potential irreversible mechanism of action. Indeed, they inactivate FAAH through covalent carbamylation of nucleophile Ser241 [54]. Currently, cyclohexylcarbamic acid 3′-carbamoyl-biphenyl-3-yl ester (URB-597), a compound that belongs to the class of carbamates, is considered one of the most potent FAAH inhibitors with an IC_50_ of 4.6 nM [34]. The main synthetic FAAH inhibitors that are being tested in clinical trials are listed in Table 1. Many of these inhibitors have passed the pre-clinical stage but have not progressed to phase 1, as reported on ClinicalTrials.gov—the official website of the U.S. Department of Health and Human Services (HHS), National Institutes of Health (NIH), National Library of Medicine (NLM), and National Center for Biotechnology Information (NCBI). An outstanding example of an inhibitor that did not pass clinical phase 1 is BIA10-2474, which, during the phase 1 trial, caused the death of one volunteer and led to neurological events ranging from mild to severe to four other volunteers. The causes of these adverse effects were found to be due to relevant off-target activity [50,55]. In general, as mentioned previously, FAAH inhibition leads to an increase in NAEs, such as AEA, which can result in anxiolytic and analgesic effects mediated by the activation of CB_1_R [40,56]. To date, synthetic inhibitors of FAAH have been extensively addressed in the literature [8,29,57]; thus, here the focus was on natural inhibitors only.

### 2.1. Natural FAAH Inhibitors

Natural FAAH inhibitors may include plants, extracts, and isolated compounds, representing a fascinating area of pharmacological research. Furthermore, due to their therapeutic applications, natural compounds are often claimed to act as nutraceuticals [58,59] and, starting from their chemical structures, they were grouped in different classes, as reported in Table 2 [49].

Several natural compounds have been identified as FAAH inhibitors (Table 2), and among them, some are pCBs [15,62]. Furthermore, natural FAAH inhibitors include kaempferol and galangin, two flavonoids; cyanidin-3-glucoside, an anthocyanin belonging to the polyphenol class; biochanin-A, an isoflavone; genistein, also an isoflavone; and daidzein, closely related to genistein (Figure 2 and Table 2).

Other flavonoids with FAAH inhibitory activity include 7-hydroxyflavone, apigenin and taxifolin. Additionally, macamide, a long-chain fatty acid *N*-benzylamide, has been studied for its potential role in FAAH modulation. Moreover, silymarin, a flavolignan extracted from milk thistle, along with its components silychristin, silydianin, silybin, and isosilybin, has been identified as a possible FAAH modulator. Lastly, 5′-methoxylicarin A and licarin A, two neolignans, and malabaricone C, a diarylnonanoid extracted from nutmeg, have been reported to interact with FAAH (Table 2).

These natural compounds, due to their structural diversity and ability to influence FAAH activity, represent an interesting alternative to synthetic inhibitors and may open new avenues in pharmacological research.

Interestingly, many of these FAAH-interacting compounds are currently undergoing clinical trials for diverse illnesses, such as epilepsy, inflammation, and menopause-related complications, as well as liver diseases (Table 3).

### 2.2. Phytocannabinoids

These is a group of more than 100 natural compounds isolated from Cannabis sativa that have been extensively studied for their pharmacological effects [9,16,62]. The most abundant pCBs, besides THC and CBD, are cannabinol (CBN) (Figure 2 and Table 2), cannabigerol (CBG), cannabichromene (CBC), and Δ^9^-tetrahydrocannabivarin (THCV) [89].

Among pCBs, CBD was found to be the most effective FAAH inhibitor, with IC_50_ values of ~10 µM for the rat enzyme [62,63]. Interestingly, CBD has been found to inhibit tumor growth in both *in vivo* and *in vitro* studies by modulating LOX activity, and hence the ECS [90]. These findings suggest a possible interaction between the oxidation and hydrolysis pathways involved in the regulation of the endogenous tone of AEA.

A dose-dependent increase in FAAH activity by CBD has also been documented in both *in vitro* and *in vivo* models [90]. Instead, CBN had IC_50_ values higher than 50 µM, and pCBs such as CBC, CBG, and THCV showed IC_50_ values higher than 100 µM towards rat FAAH [62]. In line with this, a recent study by our group investigated the ability of various pCBs to inhibit FAAH, using both *in silico* simulations and *in vitro* activity assays [31]. This study further highlighted the differences between hFAAH and rFAAH inhibition, emphasizing how enzyme structural variations may influence the efficacy of different natural compounds in modulating FAAH activity [33]. The analysis of binding free energy for various pCBs interacting with hFAAH and rFAAH revealed variations in binding affinity between the two enzymes [33]. Of note, the *in silico* data were confirmed by the *in vitro* activity assays. For instance, it was found that CBD was the most potent pCB for rFAAH inhibition, with an IC_50_ of 43.5 ± 1.5 µM, followed by CBN, with an IC_50_ of 60.0 ± 10 µM. Instead, CBC and CBG were weaker inhibitors (IC_50_ ~ 100 µM), and THCV was ineffective (IC_50_ > 100 µM). For hFAAH, all tested pCBs showed an IC_50_ > 100 µM, except for CBN, which acted as a weak inhibitor, with an IC_50_ ~ 100 µM [33].

Currently, there are 474 published clinical trials on CBD, 17 of which have reached phase 4. In contrast, only one phase 3 clinical trial on CBN is currently under investigation (Table 3).

### 2.3. Kaempferol

Kaempferol (3,5,7-trihydroxy-2-(4-hydroxyphenyl)-4H-chromen-4-one, KPF) (Figure 2 and Table 2) is a flavonoid found in Camellia sinensis, the tea plant, and is commonly present in various other plant species such as tea, spinach, broccoli, tomatoes, and grapes [67,91]. Numerous studies have highlighted the beneficial effects of KPF, including the reduction in chronic conditions like cancer, liver injury, obesity, and diabetes [67]. Additionally, it is associated with significant anti-inflammatory activity, showing potential to improve inflammation-related disorders such as intervertebral disc degeneration, colitis, and postmenopausal bone loss [67].

Notably, several studies have reported that KPF can inhibit FAAH activity [68,70]. Specifically, FAAH inhibition by KPF was evaluated at concentrations ranging from 0.1 µM to 200 µM, with the highest concentration showing an inhibition comparable to JZL195, a potent synthetic FAAH inhibitor, with an IC_50_ value of 1.064 µM on human recombinant FAAH [68]. An *in silico* study revealed that KPF interacts with the catalytic amino acids (Ser241, Phe192, Phe381, and Thr377) of the FAAH enzyme [68]. Furthermore, treatment with KPF (compared to URB597) has been linked to anxiolytic effects in fear-conditioned rats in a dose-dependent manner. This effect was abolished when co-administered with the CB1 receptor antagonist rimonabant [68].

Currently, a clinical study on KPF focused on female sexual dysfunctions, published on ClinicalTrials.gov, has completed phase 1 (Table 3).

### 2.4. Cyanidin-3-Glucoside

Cyanidin-3-glucoside (C3G) (Figure 2 and Table 2), also known as kuromanin, asterin, and chrysanthemin, is a hydrophilic compound belonging to the anthocyanin class, an important flavonoid subclass [75,76]. Anthocyanins are secondary metabolites classified within the polyphenol family and are distinguished from anthocyanidins by the presence of a glycosidic bond. In particular, C3G is characterized by a glycosylation at the C3 position [92]. Both anthocyanins and anthocyanidins have attracted scientific interest due to their role in coloring fruits, vegetables, and flowers from red to blue, as well as for health benefits such as antioxidant properties [75,76,92]. Notably, C3G has been identified as an FAAH inhibitor, with an IC_50_ value of 152.1 µM on human recombinant FAAH [75].

A clinical study is presently investigating the effects of C3G (Table 3).

### 2.5. Biochanin-A

Biochanin-A (5,7-dihydroxy-4′-methoxyisoflavone, Bc-A) (Figure 2 and Table 2) is a phytoestrogen that can be found in many plants, such as chickpeas, peanuts, and soybean [77]. Bc-A has demonstrated several beneficial effects, including slowing the progression of nonalcoholic fatty liver disease (NAFLD) by regulating cholesterol metabolism and blood lipid levels [77,93]. It also exhibits antimicrobial effects, as it appears to inhibit the growth of Clostridium, a bacterium responsible for intestinal infections, without affecting the growth of beneficial bacteria [77,94]. Furthermore, antitumoral properties have been reported, involving the downregulation of biosignaling processes related to p38, mitogen-activated protein kinase (MAPK), nuclear factor kappa-light-chain-enhancer of activated B cells (NF-kB), and protein kinase B (PKB or Akt) [77,95]. Lastly, Bc-A demonstrates significant anti-inflammatory and antioxidant effects by reducing levels of tumor necrosis factor-α (TNF-α), nitric oxide (NO), and superoxide anion, which are molecules produced in response to lipopolysaccharide (LPS) administration [77,96]. Several studies have reported that Bc-A is able to inhibit FAAH activity [79,80]. In particular, an *in silico* study revealed that it is capable of binding to Ser241 within the active site, forming a stable ligand–receptor complex with a docking score of –6.9290 [79]. To validate the results obtained *in silico*, the authors tested Bc-A in an *in vitro* assay, obtaining an IC_50_ of 2.1 ± 0.24 µM on human recombinant FAAH [79].

In keeping with the previous study, Bc-A was reported to inhibit FAAH activity in mouse brain, rat liver, and cells transfected with hFAAH, with IC_50_ values of 1.8, 1.4, and 2.4 µM, respectively [80]. Instead, Bc-A had modest effects on CB_1_ and CB_2_ receptors [80], and it was found to be selective for FAAH *versus* FAAH-2 [80]. Moreover, in an animal model of depression, treatment with Bc-A led to a significant increase in AEA levels in the prefrontal cortex, along with a significant reduction in immobility time compared to vehicle-treated controls [79]. Unfortunately, Bc-A has not yet reached the clinical phase.

### 2.6. Genistein and Daidzein

Genistein (4′,5,7-trihydroxyisoflavone, GSN) (Figure 2 and Table 2) acts as a phytoestrogen and belongs, along with biochanin-A, to the class of isoflavones [78]. Furthermore, as with many flavonoids, GSN is the aglycone form, which is less common in nature than genistin, which is the glycoside form at position 7 [97]. GSN can be found in soy-based foods, such as soy drinks and soy cheese [78]. In addition, GSN can be endogenously produced in the gut through demethylation of the methoxy group present in biochanin-A [98]. It has been reported that GSN is a strong tyrosine-specific protein kinase inhibitor of the epidermal growth factor receptor kinase [99], and this may be related to the prevention, in Asia, of the incidence of cancer diseases [97,100]. In contrast, a study on the MCF-7 cell line, which is associated with breast cancer, demonstrated that GSN has a pro-proliferative effect at low concentrations (ranging from 1.56 to 13.06 µM), whereas at higher concentrations (between 13.06 and 100 µM), it exhibits an anti-proliferative effect [101].

Furthermore, GSN, as with many isoflavonoids, shows significant antioxidant activity that can be associated with a reduction in occurrence of chronic diseases such as cardiovascular diseases by preventing the low-density lipoprotein cholesterol oxidation [102].

A recent *in silico* study revealed that GSN interacts with Ser241, achieving a docking score of −6.5791 [79]. The same authors conducted an *in vitro* FAAH inhibitory assay, which showed that GSN inhibits FAAH in a dose-dependent manner, with an IC_50_ of 1.3 ± 0.13 µM against human recombinant FAAH [79]. As reported for Bc-A, this flavone has been shown to increase AEA levels and to influence immobility time in mice [79].

According to the previous study, as reported above to Bc-A, GSN has been shown to inhibit FAAH activity in mice, rats, and humans, with IC_50_ values of 2.7, 3.1, and 4.8 µM, respectively [80].

Currently, 77 clinical trials on isoflavonoids are listed on ClinicalTrials.gov, including those categorized as recruiting, active but not recruiting, withdrawn, unknown status, terminated, and completed. Among these, five have reached phase 4 of pharmacovigilance, and four have been completed (Table 3).

Daidzein (7-hydroxy-3-(4-hydroxyphenyl)-4H-1-benzopyran-4-one, DDZ) (Figure 2 and Table 2), a derivative lacking a hydroxyl group comparable to genistein, has also been shown to inhibit FAAH activity [103,104]. DDZ is found, alongside genistein, in soy-derived foods and legumes in two different forms: daidzin, the 7-glycosilated form, releases daidzein, the aglycone form, through hydrolysis. Both can be considered analogous to human estrogens exerting protective effects against osteoporosis and cardiovascular and cerebrovascular diseases [105].

DDZ exhibits a binding affinity of −11.77 kcal/mol and a docking score of −6.2772 and it is able to interact with Ser-241 [104], while *in vitro*, DDZ has been shown to inhibit FAAH activity in mouse, rat, and human, with IC_50_ values of 4.4, 2.5, and 14 µM, respectively [80]. Furthermore, DDZ significantly reduced immobility time in the forced swim test, which is an indicator of an anti-depressive effect [104].

Notably, in Asian countries, where soy consumption is significantly higher than in Western countries, serum concentrations of genistein and daidzein can reach 2–4 μM [80,106], suggesting that the levels required to inhibit FAAH might be attainable *in vivo*, at least in theory.

Currently, there are three clinical trials that have completed phase 4 and are published on ClinicalTrials.gov) (Table 3).

### 2.7. 7-Hydroxyflavone

7-Hydroxyflavone (7-HF) (Figure 2 and Table 2) is a flavonoid found in Dracaena cochinchinensis, Clerodendron phlomoidis, and Platymiscium praecox Mart and is known to exhibit anti-nociceptive and anti-inflammatory effects [107]. An inhibitory effect of 7-HF has been demonstrated on rat FAAH, with an IC_50_ of 0.99 µM in dimethyl sulfoxide and 0.48 µM in ethanol [70]. Of note, the flavone, without or with six hydroxyl groups, does not exhibit any FAAH inhibitory activity [70]. Shifting the hydroxyl group from position 7 of 7-HF to position 5 results in the complete loss of FAAH inhibitory activity. However, adding a second hydroxyl group to 7-HF, as in the case of 3,7-dihydroxyflavone or 7,4′-dihydroxyflavone, increases the IC_50_ to 2.2 and 5.6–8.3 µM, respectively, while the addition of a hydroxyl group in positions 5 and 7, as in the case of 5,7-dihydroxyflavone (chrysin), completely abolishes the inhibitory activity. Finally, compounds such as 5,7,4′-trihydroxyflavone (apigenin) and 3,5,7-trihydroxyflavone (galangin) exhibit an IC_50_ of 35 and 31 µM, respectively, on rat FAAH [70]. Moreover, more recently, 7-HF has been reported to inhibit recombinant human FAAH, with an IC_50_ of 2.04  ±  0.19 μM [79].

Much like Bc-A and GSN, 7-HF is also able to reduce immobility time and to modulate AEA levels in animal models [79], but it has not yet reached the clinical phase.

### 2.8. Macamide

Macamides (MACs) are derivatives of non-polar and long-chain fatty acid of N-benzylamides [66] that can be found in maca (Lepidium meyenii), a central Peruvian plant [108]. MACs exhibit various pharmacological effects, such as neuroprotective, anti-fatigue, and fertility-enhancing effects [109].

The inhibitory activity of four MACs was reported, with IC_50_ values of 7.9, 7.2, and 8.5 µM for *N*-benzyl-oleamide, *N*-benzyl-linoleamide (Figure 2 and Table 2), and *N*-benzyl-linolenamide, respectively [65]. In contrast, the derivative containing a saturated fatty acid, *N*-benzyl-stearamide, exhibited lower inhibitory activity, with an IC_50_ of 43.7 µM [65]. Notably, the presence of unsaturation in the fatty acid moiety resulted in greater FAAH inhibitory activity. Moreover, mass spectrometry analysis indicates that *N*-benzyl-linoleamide is also a slow substrate for FAAH [65].

A very recent work evaluated the interaction between some MACs and rFAAH using various *in silico* techniques, finding a high affinity for two of them [108]. These latter compounds showed a reduction, albeit at high concentrations, of epileptic seizures, as well as mortality in a rat model of epilepsy [108]. It was hypothesized that this effect may be due to an interaction with FAAH.

Currently, there are 16 clinical trials published, of which 3 have reached clinical phase 3 (Table 3).

### 2.9. Silymarin

Silymarin, derived from *Silybum marianum* (L.) Gaertn (milk thistle), is a mixture of flavonolignans such as silychristins, silydianins, silybins, and isosilybins as well as the flavonoid taxifolin [71,74]. It is a nutritional supplement known for its hepatoprotective and regenerative properties. The literature also reports other effects, including antioxidant, anti-inflammatory, anticancer, antiviral, antidiabetic, immunomodulatory, and anti-arthritic activities [110,111]. Furthermore, milk thistle is used in various liver diseases, such as alcoholic liver disease, cirrhosis, hepatitis, liver fibrosis, and liver tissue regeneration [110].

A molecular docking study provides insight into the binding affinities of silybin A and B, silydianin, silychristin, isosilybin A and B, and taxifolin to FAAH, reporting the following binding free energies: −66.08, −66.67, −42.16, −57.44, −64.15, −65.68, and −42.33 kcal/mol againest rFAAH, respectively [74]. They identified that silybin A exhibits the best-predicted affinity with rFAAH and has the highest binding affinity compared to the other compounds. In fact, Silybin A penetrates deeply into the catalytic triad and interacts directly with Ser241 [74]. Moreover, the authors report the IC_50_ values of the tested inhibitors on hFAAH as follows: taxifolin, 7.20 ± 0.31 µM; silychristin, 6.11 ± 1.03 µM; silydianin, 5.38 ± 0.31 µM; silybin (Figure 2 and Table 2), 5.08 ± 0.08 µM; and isosilybin, 6.18 ± 0.38 µM [74].

The treatment with a gel formulation containing AEA and silybin against peripheral neuropathic pain is particularly interesting. Indeed, the AEA–silybin gel showed a maximum antinociceptive activity > 50% higher than AEA alone and higher than the positive control (gabapentin gel). These results indicate that FAAH inhibition improves the analgesic efficacy of AEA in peripheral neuropathic pain conditions [74].

Currently, there are 76 studies on ClinicalTrials.gov, and 13 have reached phase 4 (Table 3).

### 2.10. Nutmeg Phenols

Nutmeg is the seed of the aromatic evergreen *Myristica fragrans*, native to Indonesia, India, South Africa, and United States. This seed is widely used as a spice and in traditional medicine [85,112,113,114,115]. The significant increase in the use of nutmeg has stimulated research interest in its bioactive compounds. To date, 35 lignans and 91 neolignans, 8 diphenylalkanes, 17 phenylpropanoids, and some terpenes have been isolated from *Myristica fragrans* [115,116]. These compounds have been shown to possess antioxidant, anti-inflammatory, antibacterial, and analgesic properties [85,112,113,114,115,116]. Also, a potent hepatoprotective effect was reported after treatment with nutmeg extracts [116]. Specifically, under conditions of acute liver injury, plasma levels of alanine transaminase and aspartate transaminase were reduced 24 h after treatment with nutmeg extract (250 mg/kg), suggesting that this extract may help mitigate this condition in mice [116]. Conversely, exposure to high doses (1 and 4 g/kg) of nutmeg powder for 7 and 14 days induced an increase in the aminotransferases in mice in a dose and time-dependent manner [117]. These results suggest that nutmeg has protective effects at low concentrations, while it becomes toxic at high ones. Moreover, concentrations higher than 4 g/kg led to hypoactivity, unstable gait, and dizziness, while the LD_50_ in mice was 5.1 g/kg [113].

Nutmeg has been linked also to various neurological activities. Currently, the use of this seed has expanded beyond its traditional role as a spice, and now it is used as a recreational drug due to the hallucinogenic effect of some of its compounds [118,119]. Because of these psychoactive effects, nutmeg has also been associated with cases of overdose [119,120], as illustrated by the case of a 20-year-old man who was hospitalized after ingesting 75 g of nutmeg powder [119].

In keeping with these data, the neuropharmacological effects of different nutmeg extracts, administered orally and intraperitoneally, were compared to those of THC, amphetamine, and morphine [121]. The observed activities were not cannabinoid-like in nature and varied depending on the type of extract and the route of administration [121]. Furthermore, no binding activity was found for different nutmeg fractions relative to various CNS receptors, including CB_1_R e CB_2_R [118]. Conversely, the same fractions exerted significant inhibition of FAAH and MAGL [118]. Subsequently, 13 compounds were isolated from nutmeg [85], including myristicin, which is potentially toxic [122]. The compounds that most strongly inhibited hFAAH activity are 5′-methoxylicarin A, licarin A, and malabaricone C, with IC_50_ values of 4.57 ± 0.66, 7.02 ± 2.02, and 38.29 ± 6.18 μM, respectively [85]. The same compounds were also tested as MAGL inhibitors, without significant effects [85]. Furthermore, a significant anxiolytic effect induced by 5′-methoxylicarin A was also observed at a concentration of 120 mg/kg in mice, with no interactions with the locomotor system [85]. Taken together, these results show an indirect modulation of the ECS through FAAH inhibition, along with a notable anxiolytic effect *in vivo* [85].

No clinical trials have been yet reported on nutmeg components.

## 3. Discussion

Due to the relevance of the eCB signaling in various physio-pathological conditions [1,2,3,4,5,6,7,8,9], and to the growing interest towards natural substances (particularly those of plant origin), many compounds are being tested on FAAH, one of the best-studied ECS components.

It is important to emphasize that signaling driven by eCBs—AEA included—can be modulated through various mechanisms, such as blocking its cellular transport or modulating FAAH activity and expression [123,124].

Although a significant body of literature exists regarding the effects of compounds that activate cannabinoid receptors (CBRs) and their associated psychotropic effects, the transition from cannabis prohibition to medical acceptance hinges on the complex interaction of scientific progress, societal views, and the regulatory environment surrounding legalization.

To overcome this issue, FAAH has emerged as a promising target for modulating eCBs signaling. Thus, numerous synthetic inhibitors have been developed, many of which demonstrated potent *in vivo* effects, including anti-inflammatory and analgesic properties [29]. Unfortunately, in 2016, a synthetic FAAH inhibitor, BIA 10-2474, caused the most serious incident ever recorded during a clinical trial targeting the ECS [50,55].

It is necessary to emphasize that this episode is unique and unfortunately has represented a setback in the research, for a period, of compounds capable of interacting with FAAH. To highlight, this episode led the EMA to make changes to its guidelines (https://www.ema.europa.eu/en/news/revised-guideline-first-human-clinical-trials (accessed on 25 July 2017); https://www.fda.gov/drugs/drug-safety-and-availability/fda-finds-drugs-under-investigation-us-related-french-bia-10-2474-drug-do-not-pose-similar-safety (accessed on 12 August 2016)) [125]. Furthermore, separately, the Food and Drug Administration (FDA) determined that BIA 10-2474 exhibits a unique toxicity that does not apply to other drugs within the same class [125].

Given the potential benefits and safety profile of natural compounds, research has shifted to studying these substances as a promising strategy to modulate FAAH activity.

The completion of phase 1 clinical trials indicates a level of safety for most of the compounds discussed in this review (Table 3). Moreover, in line with this, as shown in Table 3, 35 studies have reached Phase 4, a crucial stage of a clinical trial that facilitates the collection of data on market-approved drugs, enhancing the understanding of their benefits, previously unidentified risks, and optimal use for long-term patient treatment. In this review, several natural compounds capable of inhibiting FAAH are described, whose biological effects may be indeed linked to their inhibitory power. In particular, various non-psychoactive pCBs have been shown to inhibit FAAH, but with significant species-specific selectivity [33,41].

Notably, an increase in AEA levels has been reported in the plasma and serum of CBD-treated schizophrenic patients [63,126]. Specifically, CBD has demonstrated the ability to alleviate psychotic symptoms associated with schizophrenia when compared to current medications, indicating that the capacity of CBD to inhibit FAAH activity and augment intrinsic AEA signaling may represent a functionally relevant component of its antipsychotic properties [63]. Moreover, CBD has proven effective for the treatment of cannabis use disorder compared to placebo [126].

Furthermore, several isoflavones, such as GSN, DDZ, and Bc-A, have been shown to exert an anti-depressant effect by inhibiting the FAAH-mediated degradation of AEA [79,104].

Of note, structural analysis highlights that compounds containing a rigid ring and an aromatic system have a greater ability to interact with FAAH (Figure 2). These structural remarks can also be observed in synthetic inhibitors [29]. Moreover, by analyzing the potency of the natural inhibitors presented here—excluding pCBs and C3G—it emerges that they can inhibit FAAH within a range of 1 to 15 µM. CBD and CBN exhibit greater affinity for rFAAH compared to hFAAH [33]. In particular, the *in silico* analysis showed that, despite the two isoforms being similar, they display some structural differences in the catalytic site region, making hFAAH less sensitive to the inhibitory action of these pCBs [33]. For C3G, its lower potency may be due to the ability of FAAH to primarily hydrolyze lipophilic compounds. Consequently, a compound containing the polar saccharinic residue could negatively interact with the residues present in its three functional channels and with the catalytic triad.

The research on the potential of natural compounds as inhibitors towards different targets arises from a combination of factors. One of the most significant and intriguing aspects is that, in several cases, their use has been documented through sometimes millennia-old practices, although often not according to the regulations or standards required by today’s control systems. This makes these substances particularly interesting regarding the safety of their use. Moreover, the accumulation of scientific literature over the past few decades supports the notion that regular consumption of fruits and vegetables, rich in natural substances, can exert a protective effect against the development and progression of various disorders [127,128].

Additionally, it is important to point out that even naturally derived compounds can present side effects, meaning “natural” is not equivalent to “safe” and that bioavailability studies are necessary to assess their therapeutic impact from “natural” exposure through diet.

This aspect, together with the use of new techniques (such as computational analysis), enables the screening of compound libraries at a very low cost, similar to what already happens for synthetic inhibitors [129,130]. Moreover, by analyzing the interactions and chemical structures of natural compounds, a structure–activity relationship (SAR) can be developed, providing a valuable tool for future drug design.

An additional benefit to consider is that using natural sources can reduce the environmental impact of research.

This review supports the use of natural compounds as therapeutic agents to modulate ECS. Notably, as a further advantage, FAAH inhibition represents an emerging physiological target for the treatment of several pathological conditions, including neurodegenerative diseases, inflammation, and reproductive system disorders (Figure 1) [1,2,3,4,5,6,7]. Consequently, remarkable efforts have been made both by academia and pharmaceutical companies into developing potent, selective, and safe FAAH inhibitors.

## Figures and Tables

**Table 1:** Main synthetic FAAH inhibitors and their associated clinical trials.

Compound	ID Study	Max Phase	Class	Type	IC_50_	Pathology	References
URB-597	NCT00916201	1	Carbamate	Covalent Irreversible	rFAAH (rBM): 5 nM; hFAAH (hLM): 3 nM	Schizophrenia	[34,42,44,45]
SSR411298	NCT01439919, NCT00822744	2	Carbamate	Reversible	mFAAH: 62.5 ± 8.4 nM	Persistent cancer pain and major depressive disorder	[44,46]
PF-04457845	NCT01618656, NCT02216097, NCT03386487, NCT00981357	2	Urea	Covalent Irreversible	hFAAH: 7.2 ± 0.63 nM rFAAH: 7.4 ± 0.62 nM	Cannabis dependence, post traumatic stress disorder, cannabis use disorders, and osteoarthritis	[47,48]
JNJ-42165279	NCT03664232, NCT02432703, NCT02498392	2	Urea	Covalent Reversible	hFAAH: 70 ± 8 nM rFAAH: 313 ± 28 nM	Autism spectrum disorder, phobic disorders, depressive disorder/anxiety	[47,49]
BIA 10-2474	NCT03943017	/	Urea	Covalent Irreversible	hFAAH (HEK293T): 50 to 70 nM	Health knowledge, attitudes, and safety evaluation	[50]
OL-135	/	/	α-Ketoheterocycle	Covalent Reversible	hFAAH: 15 nM	/	[34,42,44,47,51]
SA-57	/	/	Carbamate	Covalent Irreversible	hFAAH: 1–3 nM mFAAH: 1–3 nM	/	[52]

HEK293T: human embryonic kidney 293 cells; hFAAH: human FAAH; hLM: human liver microsomes; JNJ-42165279: *N*-(4-chloropyridin-3-yl)-4-[(2,2-difluoro-1,3-benzodioxol-5-yl)methyl]piperazine-1-carboxamide; mFAAH: mouse FAAH; rBM: rat brain membranes; rFAAH: rat FAAH; PF-04457845: redafamdastat; SSR411298: 2-amino-2-oxoethyl(3-((2r,5r)-5-(6-methoxynaphthalen-1-yl)-1,3-dioxan-2-yl)propyl)carbamate; URB-597: cyclohexylcarbamic acid 3′-carbamoyl-biphenyl-3-yl ester.

**Table 2:** Structural classification and potency of major natural FAAH inhibitors.

Compound	Class	Group	IC_50_	References
CBD	Terpenophenol	Phytocannabinoid	rFAAH 15.2 ± 3.2 μM rFAAH 43.5 ± 1.5 µM rFAAH 8.6 ± 0.2 µM hFAAH > 100 µM	[33,57,60,61,62,63]
CBN			rFAAH > 50 μM rFAAH 60.0 ± 10 µM hFAAH ~ 100 µM	[33,61,62]
***N***-benzyl-oleamide	*N*-benzylamide	Macamide	hFAAH 7.9 µM	[57,64,65]
***N***-benzyl-linoleamide			hFAAH 7.2 µM	[57,64,65]
***N***-benzyl-linolenamide			hFAAH 8.5 µM	[57,64,65,66]
Kaempferol	Flavonoid	Flavonol	hFAAH 1.064 µM	[67,68]
Galangin			rFAAH 31 μM	[69,70]
Taxifolin		Flavanone	hFAAH 7.20 ± 0.31 µM	[71,72,73,74]
Cyanidin-3-glucoside		Anthocyanin	hFAAH 152.1 µM	[75,76]
Biochanin-A		Isoflavone	hFAAH 2.1 ± 0.24 and 2.4 μM mFAAH 1.8 μM rFAAH 1.4 μM	[21,77,78,79,80]
Genistein			hFAAH 1.3 ± 0.13 and 4.8 µM mFAAH 2.7 µM rFAAH 3.1 µM	[22,78,79,80]
Daidzein			hFAAH 14 µM mFAAH 4.4 µM rFAAH 2.5 µM	[78,80,81]
7-Hydroxyflavone		Flavone	rFAAH 0.99 μM (DMSO) and 0.48 μM (EtOH) hFAAH 2.04 ± 0.19 μM	[70,79,82]
Apigenin			rFAAH 35 μM	[83]
Silychristin	Flavonolignans	Silymarin	hFAAH 6.11 ± 1.03 µM	[74,84]
Silydianin			hFAAH 5.38 ± 0.31 µM	[74,84]
Silybin			hFAAH 5.08 ± 0.08 µM	[57,71,74,84]
Isosilybin			hFAAH 6.18 ± 0.38 µM	[57,74,84]
5′-methoxylicarin A	Neolignan	Nutmeg	hFAAH 4.57 ± 0.66 µM	[85]
Licarin A			hFAAH 7.02 ± 2.02 µM	[85,86,87]
Malabaricone C	Diarylnonanoids		hFAAH 38.29 ± 6.18 µM	[85,88]

hFAAH: human FAAH; mFAAH: mouse FAAH; rFAAH: rat FAAH.

**Table 3:** Main natural FAAH inhibitors and their associated clinical trials.

Compound	ID Study	Status	Max Phase	Participant Group	Treatment	Outcome
Phytocannabinoids	NCT05044819	Active, not recruiting	4	Cannabidiol	CBD (100 mg/mL)	Change in fibrosis and number of participants, as assessed by an independent adjudication committee
	NCT05649059	Not yet recruiting	4	Cannabidiol	Epidiolex (3 mL) (CBD 100 mg/mL)	Change in subjective anxiety
				Placebo	Placebo (3 mL)	
	NCT03891264	Terminated	4	Cannabidiol	Epidiolex (CBD 100 mg/mL)	Changes in brain positron emission tomography signal
	NCT04899050	Completed	4	Cannabidiol	Epidiolex (CBD 20 mg/kg two day)	Number of epileptiform activities
	NCT05864846	Recruiting	4	Cannabidiol	Epidiolex (CBD 100 mg/mL)	Changes in problematic behavior severity, neuropsychiatric disorder assessment, behavior (aberrant, child, adult, and self-reported), patient-reported outcomes, sleep characteristics, executive function, caregiver-reported quality of life and family functioning, overall quality of life, symptom severity perception, retention rate, number of treatment responders, seizure frequency, suicidal ideation and attempts, number of epilepsy-releated hospitalizations, and number of withdrawals due to treatment-emergent adverse events
	NCT05022186	Unknown status	4	Cannabidiol	CBD oil 5%	Changes in cognitive functions, verbal fluency, dementia assessment, memory performance, executive function, mood and anxiety, and cerebrospinal fluid biomarkers
				Homotaurine	Vivimind (homotaurine)	
				Control	No treatment	
	NCT04749628	Completed	4	Placebo	Placebo	Change in total opioid consumption after bilateral total knee arthroplasty
				Cannabidiol 400 mg	Epidiolex (cannabidiol) (400 mg)	
				Cannabidiol 800 mg	Epidiolex (cannabidiol) (800 mg)	
	NCT04603391	Completed	4	CBD first, followed by placebo	Epidiolex (100 mg/mL, 7.5 mL) (CBD 750 mg) twice a day for three days, then CBD (750 mg) and ritalin (10 mg) on the fourth day, followed by placebo (7.5 mL) twice a day for three days, then placebo (7.5 mL) and ritalin (10 mg) on the fourth day	Changes in the GMR of Cmax will be compared between the two exposure conditions: methylphenidate + CBD vs. methylphenidate + placebo, and differences in the GMR of the AUCinf for methylphenidate will be compared between the two exposure conditions, specifically methylphenidate with CBD *versus* methylphenidate with placebo.
				Placebo first, followed by CBD	Placebo (7.5 mL) twice a day for three days, then placebo (7.5 mL) and ritalin (10 mg) on the fourth day, followed by epidiolex (100 mg/mL, 7.5 mL) (CBD 750 mg) twice a day for three days, then CBD (750 mg) and ritalin (10 mg)	
	NCT05324449	Recruiting	4	Treatment	Cannabidiol (100 mg/mL)	Changes in the CGI-I score for anxiety from baseline in pediatric epilepsy
	NCT04607603	Completed	4	Cannabidiol	CBD 3 caps/day of 200 mg	Change in WOMAC pain score
				Placebo	Placebo 3 caps/day	
	NCT04133480	Withdrawn	4	Cannabidiol	GWP42003-P (CBD 100 mg/mL)	Change in processing speed on the National Institutes of Health Toolbox Cognition Battery in pediatric patients with Lennox–Gastaut syndrome
	NCT05209867	Completed	4	Cannabidiol	CBD (63 mg/day)	Variations in the number of inflammation-related genes in PBMCs, with a significant change in expression before and after CBD treatment
	NCT04396730	Completed	4	Placebo first, followed by CBD	Contraceptives along with placebo, followed by contraceptives and CBD oil (400 mg)	Changes in maximum plasma ethinyl estradiol and concentration of levonorgestrel
				CBD first, followed by placebo	Contraceptives along with CBD oil (400 mg), followed by contraceptives and placebo	
	NCT04732169	Withdrawn	4	Cannabidiol	Different concentration of epidiolex (CBD 250 mg/day, 500 mg/day and 1000 mg/day)	Changes in Hamilton Depression Rating Scale-17
				Placebo	Placebo	
	NCT04989413	Unknown status	4	Cannabinoids	CBD (133 mg) + CBG (66 mg) + THC (4 mg)	Reduction in migraine days
				Placebo	Placebo oral drops	
	NCT04768478	Withdrawn	4	Cannabidiol	CBD (25 mg) three times a day with routine post-operative pain management	Changes is score on pain VAS and level of nausea using VAS after ankle and tibia fracture
				Control	Placebo three times a day with routine post-operative pain management	
	NCT04997954	Active, not recruiting	4	Cannabidiol oil	MediCabilis, which provides CBD + CBDA (50 mg) and THC (<2 mg)	Evaluate safety and tolerability
	NCT05961501	Not yet recruiting	3	Study group	Solution of CBD and CBN	Changes in muscle pain assessed using the PPT with the Wagner pain test FPX 25 algometer and electrical activity and nerve conduction in muscles assessed using EMG
				Placebo group	Aqueous solution	
Kaempferol	NCT06060691	Completed	1	Kaempferol	Kaempferol vaginal gel	Change in FSFI
				Placebo	Vaginal plain formulation	
Cyanidin-3-glucoside	NCT04404218	Unknown status	2	Açaí extract	Açaí extract capsule (gallic acid, catechin, chlorogenic acid, caffeic acid, p-coumaric acid, epicatechin, orientin, cyanidin-3-glucoside, luteolin, and apigenin) (520 mg) 3 times a day	Comparison of the two groups based on the 7-Point Ordinal Symptom Scale in patients with COVID-19
				Placebo	Placebo caps 3 times a day	
Genistein	NCT02796794	Unknown Status	4	Genistein	Genistein (60 mg/day) in addition to their enteral nutrition	Changes in serum TNF-α, interleukin 1-β, interleukin-6, and high mobility group box 1 in sepsis
				Control	Enteral nutrition only	
	NCT03167827	Completed	4	Isoflavone and exercise	Isoflavones 1 capsule/day (100 mg composed of 3.3% genistein, 93.5% daidzein and 3.2% glycitein) + aerobic and resistance training program	Changes in vaginal squeeze pressure, muscle function, and electromyography pelvic floor
Genistein and Daidzein	NCT01497977	Completed	4	Placebo and exercise	1 capsule/day of corn starch + aerobic and resistance training program	Change from baseline of serum lipids in postmenopausal women
				Red clover phytoestrogens	Biokain A (23 mg), daidzein (1 mg), formononetin (15 mg), and genistein (1 mg)	
				No drugs	No treatment	
	NCT02026518	Completed	4	Soy	Soy isoflavones (40 mg/day as 2 capsules/day) + placebo similar to 50,000 IU cholecalciferol	Changes in sensation of pain, flatulence, diarrhea, and constipation in patients with irritable bowel syndrome
				Soy-cholecalciferol	Soy isoflavones (40 mg/day as 2 capsules/day of genistein, daidzein and glycitin) + supplement of cholecalciferol	
				Cholecalciferol	Placebo similar to soy isoflavones (2 capsules/day) + 50,000 IU cholecalciferol	
				Placebo	Placebo in similar form of cholecalciferol + soy isoflavones	
	NCT01048606	Completed	4	Placebo and exercise	4 placebo capsules/day + aerobic and resistance exercise session	Changes in body composition, plasma lipid profile (apolipoproteins, cholesterol HDL and LDL, and triglycerides), glucose metabolism, markers of oxidative stress, quality of life, plasma fibrinogen levels
				Phytoestrogens without exercise	Isoflavones (70 mg/day composed of 44 mg of daidzein, 16 mg of glycitein, and 10 mg of genistein) 4 capsules/day	
				Phytoestrogen + exercise Placebo without exercise	Isoflavones (70 mg/day composed of 44 mg of daidzein, 16 mg of glycitein, and 10 mg of genistein) 4 capsules/day + aerobic and resistance exercise session 4 placebo capsules/day	
Macamide	NCT00181961	Completed	3	Maca root 1	Maca root (1500 mg)	Change in sexual dysfunction inventory score
				Maca root 2	Maca root (3000 mg)	
	NCT00568126	Completed	3	Maca root	Maca root (3 g/day)	Proportion of participants in remission according to ASEX and proportion of participants in remission according to MGH-SFQ
				Placebo	Inactive placebo	
	NCT00575328	Terminated	3	Maca root	Maca root (3 g/day)	ASEX and decreases in MGH-SD
				Placebo	Inactive placebo	
Silymarin	NCT03130634	Completed	4	FOLFIRI + Silymarin	FOLFIRI + silymarin (150 mg), 3 times/day	Gastrointestinal-related adverse events
				Only FOLFIRI	FOLFIRI	
	NCT04816682	Completed	4	LAGOSA (silymarin)	Silymarin tablets T.I.D 3-2-2 (150 mg/tablet)	Changes of at least one point in the COVID-19 stage and enhancement in aminotransferase activity
				Control	No treatment	
	NCT05099601	Unknown status	4	Silymarin	Silymarin topical cream (0.7%) twice daily	Compare the effectiveness of topical silymarin alone *versus* its combination with microneedling in the treatment of melasma
				Silymarin + microneedling	Silymarin topical cream (0.7%) twice daily + three sessions of microneedling	
	NCT05666765	Unknown status	4	3 experimental groups: acne vulgaris group 1, 2, and 3. The patients in each group are randomly assigned to each treatment	Isotretinoin (20 mg/day)	Change in Global Acne Grading Classification
					Silymarin (140 mg/day)	
					Isotretinoin (20 mg/day) + silymarin (140 mg/day)	
	NCT04434404	Completed	4	Placebo	Anthracycline-containing chemotherapy (50 mg/m^2^)	The addition of L-carnitine could extend the continuous administration of anthracycline-containing chemotherapy
				L-Carnitine	L-carnitine (3 tabs of 500 mg/tablet) + anthracycline-containing chemotherapy (50 mg/m^2^)	
				Silymarin	Silymarin (140 mg/capsule) + anthracycline-containing chemotherapy (50 mg/m^2^)	
	NCT02347319	Completed	4	Pennel	Garlic oil (50 mg) + DDB	Restore ALT levels to physiological values in patients with chronic liver disease
				Legalon	Silymarin (140 mg)	
				Placebo	Placebo	
	NCT04490967	Unknown status	4	A single group of patients using one treatment on one side of the face and the other treatment on the other side	On the left side: silymarin cream (1.4%), 2 times/day	Change in number of total lesions, inflammatory lesions, and non-inflammatory lesions, and evaluation of tolerability
					On the right side: salicylic acid peeling (30%), every 2 weeks	
	NCT02973295	Withdrawn	4	Silymarin	Silymarin (100 mg/cap of 2 × 200 mg) 8 weeks and 2 × 100 mg 16 weeks)	Reduction in liver steatosis parameters, assessed by CAP and liver fibrosis by LSM
				Placebo	Placebo capsule (2 × 2 caps 8 weeks and 2 × 1 caps 16 weeks)	
	NCT00412763	Completed	4	Silymarin	Silymarin	Assessment of symptoms and signs of acute hepatitis
	NCT05042245	Unknown status	4	Ornithine aspartate	Ornithine aspartate (3 g) three times a day + silymarin placebo (140 mg) 2 times a day	The percentage of patients with NAFLD whose CAP value normalized of changed by more than 10%
				Silymarin	Silymarin (140 mg) 2 times a day + ornithine aspartate placebo (3 g) three times a day	
	NCT02669641	Unknown status	4	Steatosis group	Silymarin (150 mg/cap) + Phyllanthus niruri (225 mg/cap) + choline (60 mg/cap)	Assessment of MRI spectroscopy in the quantification of fat fraction in patients with hepatic steatosis
	NCT06724952	Not yet recruiting	4	Silymarin	Methotrexate intramuscularly or subcutaneously + one tab of silymarin (140 mg/cap), once a day	Assessment of DAS-28-CRP for evaluating rheumatoid arthritis disease activity
				Placebo	Methotrexate intramuscularly or subcutaneously + one cap of placebo, once a day	
	NCT05849558	Recruiting	4	Ursoplus + silymarin	Ursodeoxycholic acid (250 mg) + silymarin (140 mg), 2 caps/12 h	Change in total serum bilirubin and direct serum bilirubin, as well as alterations in AST and ALT
				Ursodeoxycholic acid	Ursodeoxycholic acid (250 mg), 2 caps/12 h	
				Placebo	Placebo 2 caps/12 h	

ALT: alanine aminotransferase; ASEX: Arizona Sexual Experience Scale; AST: aspartate aminotransferase; AUCinf: area under the time curve from zero to infinity; CAP: controlled attenuation parameter; CGI-I: clinical global impression improvement; Cmax: peak concentration; DAS-28-CRP: disease activity score-28-C-reactive protein; DDB: biphenyl dimethyl dicarboxylate; EMG: electromyography; FSFI: female sexual function index; GMR: geometric mean ratio; HDL: high-density lipoprotein; LDL: low-density lipoprotein; LSM: liver stiffness measurements; MGH-SD: Massachusetts General Hospital Sexual Dysfunction; MGH-SFQ: Massachusetts General Hospital Sexual Functioning Questionnaire; MRI: magnetic resonance imaging; NAFLD: non-alcoholic fatty liver disease; PBMCs: human peripheral blood mononuclear cells; PPT: pressure pain threshold test; TNF-α: tumor necrosis factor-α; VAS: visual analog scale; WOMAC: Western Ontario and McMasters Universities osteoarthritis index.

## Data Availability

Not applicable.
